# Illustrations of the Botany and Other Branches of the Natural History of the Himalayan Mountains, and of the Flora of Cashmere

**Published:** 1835-01-01

**Authors:** 


*Illustrations of the Botany and other Branches of the Natural History of the Himalayan Mountains, and of the Flora of Cashmere.* By J. Forbes Royle, Esq., F.L.S., &c. Part IV. London, 1834. Folio, pp. 32.

Mr. Royle's work on Indian botany is one of those the appearance of which we scarcely know whether to regret or hail; for the magnificence of the plates must circumscribe its sale, and confine its perusal very much more than could be desired, as it contains matter of general interest and importance. The execution of the figures is admirable: some of them are drawn, as we presume from the names attached, by native artists, and they give no contemptible idea of their skill. Each number contains ten plates, and the one before us gives representations of nineteen rare or newly discovered species. Of these, *Rheum spiciforme*, *Myricaria bracteata*, and *Lythrum Cashmerianum*, are very ornamental plants; and *Sibbaldia purpurea* and *Primula rosea* and *elliptica*, peculiarly elegant. The letterpress is critical, and indeed occasionally rather too much so to suit our taste. We had rather it had contained more of immediate local observation, and less of reference to long-published works; more copious extracts from the author's journal, and fewer from books consulted since his return. We are however grateful for what he has done; and it is the interest of his own original notes, when given, that makes us regret they are not more abundant.

The principal article in the present number contains an abridgment of the chief of our knowledge, or rather an exposition of our ignorance, respecting the plant or plants which afford the several kinds of tea: from this we shall make our extracts, as a favourable specimen of the work.

"The tea plant has been supposed to be indigenous in the mountains which separate China from the Burmese territories; but we are informed by Dr. Abel that he found a small shrub, of what is commonly considered the green variety, apparently in its natural habitat, and near no plantation, at See-chou, in the province of Kiang-see, about N. lat. 26°, where the hills were covered with pines. Thunberg states that tea grows everywhere in Japan, both naturally (*sponte*) and cultivated, on the margins of fields. One species so named is described by Loureiro as found both cultivated and in a wild state in the northern provinces of Cochin-china; and the same author describes *T. oleosa* as common about Canton, both wild and cultivated. To the kindness of Mr. Reeves I am indebted for the information that there is a species of *Thea* growing wild in the neighbourhood of Macao, which is much larger in the leaf than either the black or green tea plants.

" But it has been made a question, whether the varieties of tea known in commerce are due to difference in species, or only to differences in soil, climate, culture, and mode of preparation. The latter appears to be the opinion of Kæmpfer, Thunberg, and Siebold, as they admit of but one species of *Thea*, and is that now generally entertained. Thunberg notices two varieties of *Thea bohea*, but says they can hardly be distinguished into species. Siebold states that the variety *viridis* of *T. chinensis*, D.C., is a shrub everywhere cultivated in Japan; but the variety *bohea* he had only seen in gardens, introduced from China. From this fact one would be inclined to conclude that they were distinct; and, as all the observations were made in Japan, it is probable they all three only saw one species cultivated there, as there is reason for believing that the opinion of Linnæus that two species of *Thea* yield the teas of commerce is the more correct.

" Dr. Abel, when passing through the tea country, had little doubt of there being two species of tea-plant; but he could not at the time define the character, and was unfortunate in losing his specimens in the shipwreck of the Alceste. But he mentions that the plants from the *black* and *green* tea districts differed in the form, colour, and texture of their leaves; those of the green tea being larger, thinner, and of a lighter colour than those of the black, though growing in the same soil; these differences he also observed in a large plantation near Macao. Dr. Hooker, in the Botanical Magazine, t. 3148, has given the characters of the two species. *Thea viridis*, which is the species figured, he describes as 'a large, strong-growing, almost hardy plant, with spreading branches, its leaves three to five inches long, very broadly lanceolate, pale green, singularly waved, with the margin reflexed; the flowers large, solitary, mostly confined to the upper axil. These appear in autumn, six weeks or two months earlier than those of *T. Bohea*, which is of smaller size, with remarkably erect stiff branches; leaves not above half or two-thirds the size of the former, perfectly flat, more coriaceous, of a dark green, bearing in the axils of numerous leaves two or three flowers, which are smaller, and have a slight fragrance, and are in perfection during winter. This plant cannot withstand the frosts of an English climate.'

"Mr. Reeves, whose opinions, from his long residence in China, and attention to subjects of natural history, are entitled to the greatest weight, is the most recent author who has referred to this subject; and he expresses his surprise 'that any person who has been in China, or, indeed, any one who has seen the difference in the colour of the infusions of black and green tea, could suppose for a moment that they were the produce of the same plant, differing only in the mode of curing; particularly as they do not grow in the neighbourhood of each other.' (*Loudon's Gard. Mag.*, v. ix. p. 713.) To this opinion, it will be seen, he still adheres, as, in a letter with which I have been favored, he informs me that he believes that the *Thea viridis* of the gardens is the plant from which the green tea of commerce is prepared, and that the plant which produces the black tea of commerce, as souchong, congou, &c., is not so common in England. Both may be seen in great perfection in the Messrs. Loddige's rich and extensive nursery-grounds at Hackney, where a green-tea plant has lived for many years in the open air. The first impression on seeing them, is that of surprise at their ever having been confounded, as nothing can be more distinct, than the large, membranous, light green, wavy leaf, with large and irregular serratures, and straggling habit of the green-tea plant, from the smaller, flat, thick and coriaceous, dark green leaf, with small and even serratures, and erect port of the black tea. Both plants have been figured in Loddige's Bot. Cab. t. 226 and 227, and the characters well given, as also in the above extract from Dr. Hooker. I would only add, that the flowers, though commonly, are not always single, in the axils of *Thea viridis;* and this, though earlier in flowering, is not so much so as described. The green tea being the hardier, is cultivated, as we shall see, in the northern, and the black tea in the southern provinces of China. The former is the only kind cultivated in Japan, according to Siebold, and is that figured by Kæmpfer, *Amœn. Exot.*, p. 607.

"Notwithstanding the above opinions, and the distinctness in the characters of the two species, as above given, there is an unaccountable discrepancy in the statements, as to the plants which afford the *green* and *black* teas of commerce, especially as Dr. Abel, after giving his opinion that there were two species of tea-plant, mentions that 'from persons perfectly conversant with the Chinese method, he learnt that either of the two plants will afford the black or green tea of the shops, but that the broad thin-leaved plant is preferred for making the green tea.' (*Journ. to China*, p. 222.)

"This is in conformity with the information communicated to Dr. Hooker, and also with that originally given by Mr. Pigou (*As. An. Reg.* 1802), on the authority of a Chinese, who had been eight times in the bohea country, remaining there from four to six months each time, and who stated that 'bohea may be cured as hyson, and hyson as bohea.' To this Mr. Reeves replies, in the letter to which I have alluded, that 'the Chinese manufacturers do not, and they say they cannot, convert black tea into green, and *vice versâ:* and this I believe to be true; indeed, the colour of the infusions is alone sufficient evidence.' The discrepancy in the information Mr. Reeves explains, by adding, that 'there is a species of tea grown in the province of Canton of a pale-coloured leaf, (occasionally mixed with congou tea, to make the tea imported under the name of bohea,) and this tea can be coloured and made up to imitate various qualities of green tea, and large quantities are yearly thus made; but still it is only an appearance that can be given, the deception is detected as soon as it is put into water.' Owing no doubt to these mixtures is the difficulty in detecting the two kinds of leaf in the teas of commerce; but in good teas they may be distinctly recognised. Dr. Abel's information having been obtained from hearsay at Canton, most probably refers to the kind described by Mr. Reeves, as he most particularly distinguishes, and lays down on his map, the green and black tea districts; but, arguing upon the correctness of the information he had obtained, concludes that the differences observed may be produced by a due management of the heat used in drying the plant. Mr. Millet's account, Mr. Reeves says, he himself knows refers to some of this tea." (P. 109.)

Notwithstanding the arguments of our author, we confess ourselves still to be sceptical of the specific difference of the plants affording the black and green teas of commerce: that the variations exist between them which Mr. Royle dwells on we at once admit, but we do not think them to be more than sufficient to mark varieties of the same species. This opinion is supported by the fact, that the black and green trees are grown on different districts, as well as by the assertion made by the Chinese, that if green-tea plants be taken to the black-tea districts, they will produce, not green, but black tea, and *vice versâ*. The leaves of both kinds, when unrolled, are very similar in general characters; and this leads us to observe, in conclusion, that, however much doubt may rest on the subject, there is no doubt that the plant here grown as the black-tea plant, and called *Thea Bohea*, is not the plant that yields the black or bohea teas of China: it is totally and specifically distinct.

